# Impact of Sex on Outcomes With Femoral Artery Closure Devices Versus Manual Compression in Patients Undergoing Percutaneous Coronary Intervention

**DOI:** 10.1002/hsr2.70256

**Published:** 2024-12-19

**Authors:** Wesley L. Anderson, Asad J. Torabi, Brian A. O'leary, Jeffrey A. Breall, Anjan K. Sinha, Ziad A. Jaradat, Michelle C. Morris, Kyle A. Frick, Ibrahim A. Romeh, Ali F. Iqtidar, Elisabeth von der Lohe, Rolf P. Kreutz

**Affiliations:** ^1^ Department of Medicine, Division of Cardiovascular Medicine Indiana University School of Medicine Indianapolis Indiana USA

**Keywords:** bleeding, cardiac catheterization, percutaneous coronary intervention, sex, women, vascular closure devices

## Abstract

**Background and Aims:**

Femoral artery access is widely used despite recent increase in radial access for percutaneous coronary interventions (PCI). Femoral artery closure devices are used to shorten vascular closure time and reduce bleeding. We sought to examine sex‐based outcomes of femoral artery vascular closure devices (VCD) in patients undergoing PCI.

**Methods:**

We identified patients who had undergone PCI (*n* = 11,415) in the Indiana University Health Multicenter Cardiac Cath registry using femoral artery access. Clinical outcomes were compared between VCD and manual compression and analyzed according to sex. Patients with cardiogenic shock and left ventricular support devices were excluded.

**Results:**

The use of any vascular closure device as compared to femoral artery manual compression was associated with a reduction in 72‐h bleeding events (adjusted odds ratio [OR]: 0.64; 95% confidence interval [CI]: 0.46–0.87). With manual compression, women had higher rates of 72‐h bleeding as compared to men (4.5% vs. 1.6%, *p* < 0.001). Women demonstrated greater absolute risk reduction in 72‐h bleeding events with use of VCD as compared to men (2.8% vs. 0.8%, *p* < 0.001). For women, VCD were associated with lower risk of access site bleeding (OR: 0.43; 95% CI: 0.24–0.8), hematoma (OR: 0.36; 95% CI: 0.2–0.63), and vascular complications (OR: 0.25, 95% CI: 0.09–0.72). Use of VCD was associated with lower risk of in‐hospital death (adjusted OR: 0.4; 95% CI: 0.28–0.58; *p* < 0.001) in multivariable regression analysis.

**Conclusion:**

Women derive more benefit from use of femoral artery VCD during PCI than men with greater reduction in bleeding rates, access site hematoma, and vascular complications.

**Abbreviations**
CI: confidence interval
ESRD: end stage renal disease
Hgb: hemoglobin
NCDR: National Cardiovascular Data Registry
OR: odds ratio
PCI: percutaneous coronary intervention
VCD: vascular closure device

## 1 Introduction

Bleeding and vascular complications in the setting of percutaneous coronary intervention (PCI) have a large effect on morbidity and mortality [1, 2]. Radial artery access is known to significantly reduce bleeding events [3, 4]. However, femoral artery access remains a standard access form in various clinical scenarios. Complications of femoral access include access site bleeding, hematoma, vascular complications, and retroperitoneal bleeding. Women undergoing PCI have more bleeding events and poorer outcomes compared to men [5, 6, 7]. Bleeding events are twice as likely in women than men regardless of access approach, but this risk increases further with the femoral approach [8]. Various vascular closure devices (VCD) are available for use in the United States. Meta‐analyses have suggested similar complication rates of VCD compared with manual compression; however, there is significant heterogeneity in studies over time [9, 10, 11]. Pivotal clinical studies of VCD used to support regulatory device approval focused on device safety and early ambulation as clinical endpoints and were underpowered to assess small differences in more rare vascular complications or effect on overall mortality. In addition, at the time most prospective studies for closure devices were conducted, standard clinical practice of femoral vascular access usually did not incorporate routine use of ultrasound imaging. There is limited data on sex‐specific access outcomes with the use of current VCD in contemporary practice. We hypothesized that femoral artery closure devices are associated with improved outcomes compared with manual compression with more pronounced risk reduction in female patients.

## 2 Methods

### 2.1 Objective

The objective of this study was to examine rates of complications and clinical outcomes between men and women who underwent PCI using femoral artery access and to compare vascular access closure devices with manual compression between these groups.

### 2.2 Patient Population

Data was collected from the Indiana University Health Multi‐center Cath Registry, which included patients undergoing PCI in Indiana, USA, between 2015 and 2021 at seven participating hospitals (IU Health Methodist, West, North, Saxony, Ball, Bloomington, and Arnett hospitals). The registry includes data of all PCI cases performed at the participating institutions with clinical outcomes recorded according to standardized definitions. Institutional Review Board approval was obtained for this study (protocol# 2009681641).

### 2.3 Study Design and Endpoints

We identified patients who underwent PCI via femoral artery with closure by manual compression or device closure. Closure devices included: Angio‐Seal device (Angio‐Seal VIP; Terumo), Suture based closure (Perclose A‐T, Perclose ProGlide, Abbott), or Mynx (Mynx ACE or MYNXGRIP; Cordis). Demographics, comorbidities, clinical variables, and procedural details, were prospectively obtained as defined by the National Cardiovascular Data Registry (NCDR) Cath PCI database [12]. Patient level information on access sheath size was not available in the registry. However, all patients who presented with cardiogenic shock or had a left ventricular support device placed (intraaortic balloon pump, Impella, extracorporal membrane oxygenation) were excluded from analysis. Fluoroscopy guidance and femoral angiography is standard in our institutions to assess closure device candidacy. The use of ultrasound and micropuncture technique was variable and at the discretion of the operator. Data on these variables were not collected.

Study endpoint definitions were used as defined by NCDR CathPCI during the index hospitalization. Endpoints included access site bleeding (defined as any access site bleeding with hemoglobin [Hgb] drop ≥ 3 g/dL, blood transfusion, or requiring surgery/intervention), hematoma at access site (defined as hematoma with Hgb drop ≥ 3 g/dL, blood transfusion, or requiring surgery/intervention), retroperitoneal hemorrhage (defined as any documented retroperitoneal bleeding with Hgb drop ≥ 3 g/dL, blood transfusion, or requiring surgery/intervention), other vascular complications requiring intervention, and any bleeding event within 72 h (defined as any bleeding event with Hgb drop ≥ 3 g/dL, blood transfusion, or requiring surgery/intervention) [12]. Clinical events were collected retrospectively through review of medical records.

### 2.4 Statistical Analysis

Baseline variables were compared using Pearson *χ* ^2^ test. Continuous data were compared using analysis of variance for the various groups. Fisher's exact test was used to compare event rates. Testing was performed two‐sided with *p* < 0.05 being considered significant. Multivariate binary logistic regression models for all bleeding events and mortality were used with a forward stepwise method forcing all clinical baseline variables into a regression eliminating the least significant each step. Statistical analysis was performed with the use of SPSS software, version 28.0 (IBM Corp).

## 3 Results

We identified 11,415 subjects who underwent PCI using femoral arterial access in the Indiana University Health Multicenter Cardiac Cath Registry after excluding patients who required hemodynamic support and large bore arterial access (intra‐aortic balloon pump [≥ 7.5 F], Impella [≥ 13 F]). During the study period, radial artery access was used in 42% of PCI cases. VCD were used in 7320 (64%) of femoral artery access cases. The most used closure devices were Angioseal (*n* = 3577), suture‐based closure devices (*n* = 1464), and Mynx (*n* = 2306). As expected, clinical variables were not evenly balanced between manual compression and closure device groups. Patients who received closure deceives were more likely to be male as compared to female (67% vs. 33%), have higher body mass index (30.8 vs. 30 kg/m^2^), and exhibited lower rates of peripheral vascular (14.2% vs. 18.9%) and cerebrovascular disease (11.7% vs. 16.1%). Manual compression was more often used in patients presenting with acute coronary syndrome (42.4% vs. 28.8%) and cardiac arrest (5.4% vs. 2%) (Table 1) [13]. Differences in baseline variables between men and women are listed in Table 2.

**Table 1:** Demographics and procedural variables according to use of femoral artery manual compression vs any femoral artery closure device.

Variable	Manual compression (*n* = 4095)	Any closure device (*n* = 7320)	*p*‐value
Female	1482 (36.2%)	2435 (33.3%)	< 0.002
Male	2613 (63.8%)	4885 (66.7%)	
Age, years	66.1 ± 12.4	64.7 ± 12	< 0.001
White	3807 (93.0%)	6567 (89.9%)	< 0.001
Black	241 (5.9%)	593 (8.1%)	< 0.001
Asian	25 (0.6%)	103 (1.4%)	< 0.001
Body mass index, kg/m^2^	30 ± 9	30.8 ± 7.3	< 0.001
Diabetes mellitus	1784 (43.6%)	3266 (44.6%)	0.280
Hypertension	3383 (82.6%)	6095 (83.3%)	0.377
Hyperlipidemia	3167 (77.4%)	5818 (79.5%)	0.008
Prior stroke	658 (16.1%)	859 (11.7%)	< 0.001
Peripheral vascular disease	773 (18.9%)	1038 (14.2%)	< 0.001
Prior PCI	1799 (43.9%)	3484 (47.6%)	< 0.001
Prior CABG	874 (21.3%)	1547 (21.1%)	0.812
Chronic lung disease	791 (19.3%)	1070 (14.6%)	< 0.001
End‐stage renal disease	219 (5.3%)	421 (5.8%)	0.373
Cardiac arrest	223 (5.4%)	143 (2.0%)	< 0.001
ST‐elevation myocardial infarction	1256 (30.7%)	1385 (18.9%)	< 0.001
Non‐ST‐elevation myocardial infarction	1273 (31.1%)	2056 (28.1%)	< 0.010
Glycoprotein IIb/IIIa inhibitor	1074 (26.2%)	744 (10.2%)	< 0.001

*Note:* Values are mean ± SD or *n* (%). Abbreviations: CABG, coronary artery bypass grafting; PCI, percutaneous coronary intervention.

**Table 2:** Demographics and procedural variables compared by sex.

Variable	Men (*n* = 7498)	Women (*n* = 3917)	*p*‐value
Age, years	63.1 ± 12.4	66.1 ± 12	< 0.001
White	6899 (92.0%)	3475 (88.7%)	< 0.001
Black	447 (6%)	387 (9.9%)	< 0.001
Asian	95 (1.3%)	33 (0.8%)	0.04
Body mass index, kg/m^2^	30.8 ± 8	31.5 ± 9	< 0.001
Diabetes mellitus	3135 (41.8%)	1915 (48.9%)	< 0.001
Hypertension	6121 (81.6%)	3357 (85.7%)	< 0.001
Hyperlipidemia	5829 (77.7%)	3156 (80.6%)	< 0.001
Prior stroke	873 (11.6%)	644 (16.4%)	< 0.001
Peripheral vascular disease	1170 (15.6%)	641 (16.4%)	0.29
Prior PCI	3566 (47.6%)	1717 (43.8%)	< 0.001
Prior CABG	1777 (23.7%)	644 (16.4%)	< 0.001
Chronic lung disease	1104 (14.7%)	757 (19.3%)	< 0.001
End‐stage renal disease	413 (5.5%)	227 (5.8%)	0.52
Cardiac arrest	255 (3.4%)	111 (2.8%)	0.1
ST‐elevation myocardial infarction	1841 (24.6%)	800 (20.4%)	< 0.001
Non‐ST‐elevation myocardial infarction	2075 (27.7%)	1254 (32%)	< 0.001
Glycoprotein IIb/IIIa inhibitor	1261 (16.8%)	557 (14.2%)	< 0.001

*Note:* Values are mean ± SD or *n* (%). Abbreviations: CABG, coronary artery bypass grafting; PCI, percutaneous coronary intervention

**Figure 1:**
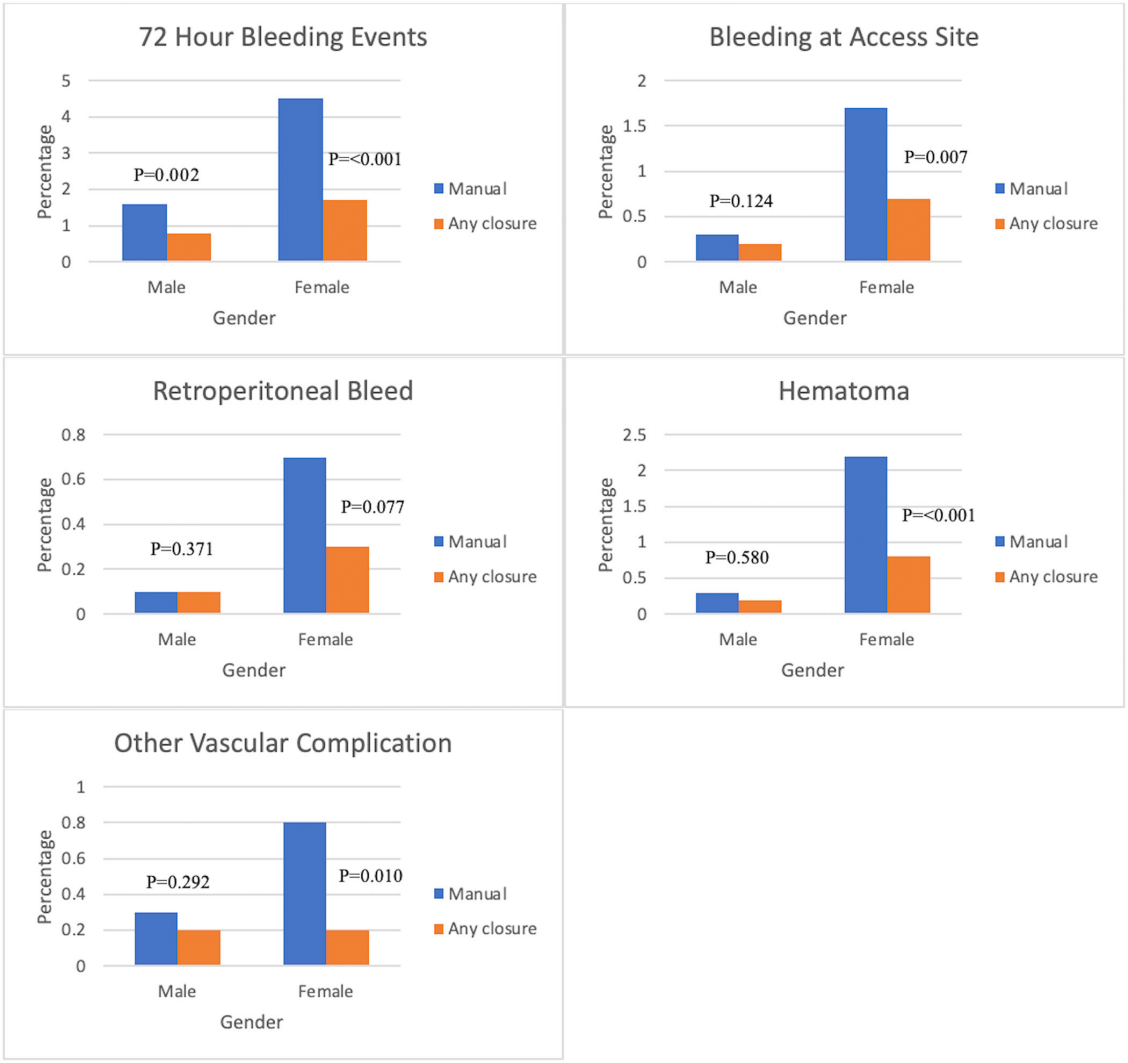
Incidence of periprocedural complications (72‐h bleeding events, hematoma, bleeding at access site, retroperitoneal bleeding, vascular complications) comparing manual compression and any vascular closure device, stratified according to sex (comparison made with Fisher's exact test).

Female patients had higher rates of bleeding and vascular complications when compared to men (Table 3). The rate of 72‐h bleeding events in women receiving manual compression was 4.5% compared to 1.6% for men (*p* < 0.001). For all patients, the use of vascular closure device was associated with reduced incidence of any 72‐h bleeding events as compared with manual compression (1.1% vs 2.1%, odds ratio [OR]: 0.42; 95% confidence interval [CI]: 0.32–0.57, *p* < 0.001). Women demonstrated a greater reduction in 72‐h bleeding events with use of VCD as compared to manual compression than men (absolute risk reduction: 2.8% vs. 0.8%; *p* < 0.001) (Table 3). The risk of access site bleeding (1.7% vs. 0.3%, *p* < 0.001), hematoma (2.2% vs. 0.3%, *p* < 0.001), and retroperitoneal bleeding (0.7% vs. 0.04%; *p* < 0.001) were higher for females as compared to males with manual compression (Table 4). However, these event rates were all significantly reduced by closure devices (Tables 3 and 4, Figure 1). Among women, the use of manual compression as compared to VCD was associated with a higher risk of any 72‐h bleeding (OR: 2.54; 95% CI: 1.73–3.74, *p* < 0.001), access site bleeding (OR: 2.31; 95% CI: 1.26–4.25, *p* = 0.007), hematoma (OR: 2.8; 95% CI: 1.59–4.99, *p* < 0.001), and vascular complication (OR: 3.98; 95% CI: 1.4–11.3, *p* = 0.01). Among men, the rate of any 72‐h bleeding events was higher with manual compression as compared with use of a VCD (OR: 1.99; 95% CI: 1.28–3.09, *p* = 0.002), but the rates of access site bleeding, vascular complications, hematoma, and retroperitoneal bleeding were not significantly different. Among the various closure devices, there was no difference in any 72‐h bleeding rates between men and women when Angioseal was used (1% vs. 1.7%, *p* = 0.07). In contrast, risk of any 72‐h bleeding event was higher for women as compared to men if a suture‐based closure device (2.1% vs. 0.3%, *p* < 0.001) or Mynx device were used (1.8% vs. 0.8%, *p* = 0.048) (Table 4).

**Table 3:** Clinical access site‐related events according to manual compression versus any closure device in males and females.

Clinical events	Manual	Any closure device	Odds ratio (95% CI)	*p*‐value
			Manual versus any closure device	
Female				
Any 72‐h bleeding	66/1482 (4.5%)	44/2444 (1.7%)	2.54 (1.73–3.74)	< 0.001
Access site bleeding	25/1482 (1.7%)	18/2444 (0.7%)	2.31 (1.26–4.25)	0.007
Hematoma	32/1482 (2.2%)	19/2444 (0.8%)	2.82 (1.59–4.99)	< 0.001
Retroperitoneal bleed	11/1482 (0.7%)	8/2444 (0.3%)	2.28 (0.91–5.67)	0.077
Vascular complication	12/1482 (0.8%)	5/2444 (0.2%)	3.98 (1.40–11.3)	0.010
Male				
Any 72‐h bleeding	41/2613 (1.6%)	39/4903 (0.8%)	1.99 (1.28–3.09)	0.002
Access site bleeding	9/2613 (0.3%)	8/4903 (0.2%)	2.11 (0.82–5.49)	0.124
Hematoma	7/2613 (0.3%)	10/4903 (0.2%)	1.31 (0.50–3.46)	0.580
Retroperitoneal bleed	1/2613 (0.04%)	5/4903 (0.1%)	0.38 (0.04–3.21)	0.371
Vascular complication	8/2613 (0.3%)	9/4903 (0.2%)	1.67 (0.64–4.33)	0.292

**Table 4:** Vascular and bleeding complication rates with various closure methods for femoral artery access compared by sex.

Closure method	Male	Female	*p*‐value
Manual compression			
Any 72‐h bleeding	41/2613 (1.6%)	66/1482 (4.5%)	< 0.001
Access site bleeding	9/2613 (0.3%)	25/1482 (1.7%)	< 0.001
Hematoma	7/2613 (0.3%)	32/1482 (2.2%)	< 0.001
Retroperitoneal bleed	1/2613 (0.04%)	11/1482 (0.7%)	< 0.001
Vascular complication	8/2613 (0.3%)	12/1482 (0.8%)	0.020
Any closure device			
Any 72‐h bleeding	39/4903 (0.8%)	44/2444 (1.8%)	< 0.001
Access site bleeding	8/4903 (0.2%)	18/2444 (0.8%)	< 0.001
Hematoma	10/4903 (0.2%)	19/2444 (0.8%)	< 0.001
Retroperitoneal bleed	5/4903 (0.1%)	8/2444 (0.3%)	0.031
Vascular complication	9/4903 (0.2%)	5/2444 (0.2%)	0.846
Angioseal			
Any 72‐h bleeding	24/2411 (1.0%)	20/1166 (1.7%)	0.071
Access site bleeding	4/2411 (0.2%)	7/1166 (0.6%)	0.028
Hematoma	4/2411 (0.2%)	8/1166 (0.7%)	0.012
Retroperitoneal bleed	3/2411 (0.1%)	4/1166 (0.3%)	0.166
Vascular complication	6/2411 (0.2%)	1/1166 (0.1%)	0.302
Suture based closure			
Any 72‐h bleeding	3/1040 (0.3%)	9/424 (2.1%)	< 0.001
Access site bleeding	0/1040 (0.0%)	6/424 (1.4%)	N/A
Hematoma	0/1040 (0.0%)	2/424 (0.5%)	N/A
Retroperitoneal bleed	0/1040 (0.0%)	0/424 (0.0%)	N/A
Vascular complication	0/1040 (0.0%)	1/424 (0.2%)	N/A
Mynx			
Any 72‐h bleeding	12/1452 (0.8%)	15/854 (1.8%)	0.048
Access site bleeding	4/1452 (0.3%)	5/854 (0.6%)	0.251
Hematoma	6/1452 (0.4%)	9/854 (1.1%)	0.067
Retroperitoneal bleed	2/1452 (0.1%)	4/854 (0.5%)	0.133
Vascular complication	3/1452 (0.2%)	3/854 (0.4%)	0.511

Multivariable logistic regression was performed to assess the effect of different baseline variables on 72‐h bleeding events and death. Eight different variables were independently associated with 72‐h bleeding events, including female sex (OR: 2.6; 95% CI: 1.9–3.4, *p* < 0.001) and vascular closure device use (OR: 0.64; 95% CI: 0.46–0.87, *p* = 0.005) (Table 5). Six different variables were independently associated with in‐hospital death in multivariate regression analysis, including use of manual compression (OR: 2.5; 95% CI: 1.72–3.6, *p* < 0.001) (Table 6). No significant co‐linearity was detected on multivariable regression analysis for co‐variates.

**Table 5:** Multivariable forward conditional binary logistic regression analysis for occurrence of any 72‐h bleeding events.

Variables (any 72‐h bleeding events)	Adjusted odds ratio	95% CI	*p*‐value
Any closure device versus manual compression	0.64	0.46–0.87	0.005
Sex (male vs female)	0.39	0.29–0.529	< 0.001
Age	1.03	1.01–1.04	< 0.001
Diabetes mellitus	0.72	0.52–0.99	0.046
Glycoprotein IIb/IIIa inhibitor	1.98	1.36–2.89	< 0.001
End stage renal disease	2.95	1.72–5.05	< 0.001
Cardiac arrest	3.94	2.44–6.37	< 0.001
ST‐elevation myocardial infarction	2.08	1.44–3.01	< 0.001

**Table 6:** Multivariable forward conditional binary logistic regression analysis for occurrence of all cause in hospital death before discharge.

Variables (death)	Adjusted odds ratio	95% CI	*p*‐value
Any closure device versus manual compression	0.4	0.28–0.58	< 0.001
Age	1.03	1.02–1.05	< 0.001
Cardiac arrest	10.37	6.87–15.65	< 0.001
ST‐elevation myocardial infarction	9.74	5.56–17.08	< 0.001
Non‐ST‐elevation myocardial infarction	3.46	1.96–6.1	< 0.001
Glomerular filtration rate	0.97	0.97–0.98	< 0.001

## 4 Discussion

We have previously reported access‐related outcomes in patients undergoing cardiac catheterization for both diagnostic angiography as well as coronary intervention according to use of femoral artery closure devices [13]. In that analysis, we demonstrated a reduction in bleeding events with use of closure devices in femoral access PCI cases [13]. In our current analysis, we specifically examined sex‐based event rates with use of closure devices. Historically women undergoing PCI have been known to have higher rates of bleeding and vascular complications when compared to men, particularly with use of femoral artery access [5, 6, 7]. Our study analyzing contemporary data of femoral vascular closure methods during PCI also demonstrates that women continue to exhibit higher rates of bleeding and vascular complications as compared to men. Daugherty et al. reported two‐fold increased bleeding for women over men (7.8% vs. 3.7%, OR: 1.95, 95% CI: 1.91–2.02) [14], and Ahmed et al. and Sharma et al. showed a twofold increase in bleeding and vascular complications for women compared to men [15, 16]. Our study had overall lower bleeding rates but a similar increased risk ratio for females. The difference in bleeding rates between our study and the mentioned trials is partially due to heterogeneity in the definitions of bleeding and vascular complications, as well differences in periprocedural antithrombotic therapies with lesser use of glycoprotein IIb/IIIa inhibitors.

One randomized controlled trial evaluating VCD in women undergoing diagnostic angiography found no significant difference in access site complications when compared to manual compression [17]. However, time to hemostasis was shorter with VCD and intravascular compared to extravascular VCD was associated with a trend to reduction in access site complications [17]. Similar to the study by Gewalt et al., prior studies from our own institution have shown that VCD do not significantly reduce access site complications in diagnostic catheterization alone, particularly if smaller sheath sizes are used [13]. Patients undergoing PCI often require larger sheath sizes and routine periprocedural anticoagulation for which a VCD will offer more benefit. A large study by Farooq et al. showed a reduction in 30‐day mortality in patients treated with VCD which was slightly more evident in women (Hazard ratio: 0.85: 95% CI: 0.77–0.94: *p* = 0.037) [18]. Our study only examined patients undergoing PCI, but we excluded patients who received concomitant large bore access for ventricular assist devices which would affect incidence of vascular bleeding events. In our analysis, VCD significantly reduced the rates of all bleeding and vascular complications, except for retroperitoneal bleeding which was still numerically reduced. Women received greater benefit from VCD than men (Table 4) with the greatest absolute risk reduction seen for all 72‐h bleeding events.

Female patients have more vascular complications after PCI than men [5, 6, 7]. The reason why female patients have a predisposition for vascular complications is not clear. The increased bleeding risk in women is likely multifactorial from anatomic differences in vascular structure and the complex hormonal effects on hemostasis and the vascular bed. Women have smaller diameter arteries than men, which may limit the use of VCD in borderline diameter cases [19]. Smaller vessel diameter makes access more challenging and can result in multiple sticks that may lead to increased bleeding. This risk can be partially mitigated with ultrasound guided access and micro puncture technique. The effects of estrogen and menopausal state on the vascular wall and coagulation are complex. Menopausal status was not collected in our cohort, but the average age for women was 64.9 years. Therefore, the majority were likely postmenopausal. Due to a lack of estradiol the postmenopausal state results in vascular endothelial cell fragility [20, 21]. Autopsy studies have also shown that when compared to premenopausal women the endothelium of ascending aortas is thinner after menopause [22, 23]. There is also decreased platelet activation status after menopause and a coagulation cascade shift away from clot formation [24, 25]. In addition, the density of connective tissue encircling femoral artery vascular structures is less dense and thus less likely to contain extravasation of blood from the access puncture. All of these factors likely contribute to sex‐based differences and larger benefit seen with VCD use in female patients.

The use of collagen‐based VCD in women was previously associated with up to eightfold higher rates of vascular complications [26, 27]. This was felt to be related to smaller arterial size in women. The study by Eggebrecht et al showed symptomatic femoral artery stenosis after collagen‐based VCD to be more frequent in women than in men (0.9% vs. 0.1%; *p* = 0.077) [26]. However, the use of femoral angiography before deployment of collagen‐based VCD was not common practice in this study. In contrast, femoral angiography was performed before deployment of all collagen‐based VCD in our cohort. In our study, there was no difference between women and men in the incidence of vascular complications from collagen based VCD (0.1% vs. 0.2%; *p* = 0.302). There were higher rates of access site bleeding and hematoma in women as compared to men. However, when Angioseal was compared to manual compression these events were all reduced for female patients, and the absolute risk reduction was greater than that for men.

There is limited data on sex‐specific outcomes with extravascular sealant devices. Early safety trials for extravascular sealant devices did not report sex‐based differences in failure rates or complications [28, 29]. One trial by Noory et. al evaluating MYNX CONTROL and FemoSeal in patients undergoing peripheral vascular interventions showed that female sex was not a predictor of failure for either device [30]. A similar trial by Hwang et al. did not show sex differences in device failure but showed that low BMI was predictive of device failure [31]. Our study showed no sex differences in access site bleeding, hematoma, retroperitoneal bleed, or vascular complications for the extravascular sealant device MYNX. In our study in the multivariable logistic regression analysis, body mass index was not found to be an independent predictor of any 72‐h bleeding events; however, bleeding rates were higher for female patients than male patients. The risk of bleeding was significantly lower when compared to manual pressure and the absolute risk reduction was larger for females compared to males.

Early safety and randomized trials evaluating suture closure devices did not report sex‐based differences in failure rates or complications [32, 33, 34]. There is limited contemporary data evaluating sex‐related outcomes with these closure devices [35]. Our study showed no difference in access site bleeding, hematoma, retroperitoneal bleed, or vascular complications between males and females in the suture VCD group. Women had significantly more 72‐h bleeding events with use of VCD than men, but these events were significantly lower when compared to manual pressure and the absolute risk reduction was larger for females (2.4%) compared to males (1.3%).

There are several limitations of our study. This was a retrospective and nonrandomized multicenter registry study, and although the individual hospitals in the registry operated independently, they were part of a larger healthcare system. Patients in the manual compression group may not have been eligible for a closure device due to vascular disease or other confounders which may impact the risk of bleeding or other vascular complications. Information on the use of ultrasound and micro‐puncture technique was not collected which could have influenced the rate of vascular complications.

## 5 Conclusion

In conclusion, our study demonstrates that use of a VCD during PCI is associated with a significant reduction in access site complications when compared to manual compression. Women have higher rates of access site complications as compared to men. The reduction in access site complications was more pronounced for female patients when compared to males.

## Author Contributions

**Wesley L. Anderson:** data curation, formal analysis, investigation, validation, writing–original draft, writing–review and editing. **Asad J. Torabi:** conceptualization, writing–review and editing. **Brian A. O'leary:** conceptualization, data curation. **Jeffrey A. Breall:** conceptualization, data curation, writing–review and editing. **Anjan K. Sinha:** conceptualization, data curation, writing–review and editing. **Ziad A. Jaradat:** conceptualization, data curation, writing–review and editing. **Michelle C. Morris:** conceptualization, data curation, writing–review and editing. **Kyle A. Frick:** conceptualization, data curation, writing–review and editing. **Ibrahim A. Romeh:** conceptualization, data curation, writing–review and editing. **Ali F. Iqtidar:** conceptualization, data curation, writing–review and editing. **Elisabeth von der Lohe:** conceptualization, data curation, supervision, writing–review and editing. **Rolf P. Kreutz:** conceptualization, data curation, formal analysis, investigation, supervision, writing–review and editing.

## Ethics Statement

This trial was approved by the Indiana University Institutional Review Board.

## Conflicts of Interest

The authors declare no conflicts of interest.

## Transparency Statement

The lead author Wesley L. Anderson affirms that this manuscript is an honest, accurate, and transparent account of the study being reported; that no important aspects of the study have been omitted; and that any discrepancies from the study as planned (and, if relevant, registered) have been explained.

## Data Availability

The data that support the findings of this study are available from the corresponding author upon reasonable request.

## References

[1] M. Vaduganathan , R. Harrington , G. Stone , et al., “Short‐and Long‐Term Mortality Following Bleeding Events in Patients Undergoing Percutaneous Coronary Intervention: Insights From Four Validated Bleeding Scales in the CHAMPION Trials,” EuroIntervention 13 (2018): e1841–e1849. 28988157 10.4244/EIJ-D-17-00723

[2] R. Mehran , S. Pocock , E. Nikolsky , et al., “Impact of Bleeding on Mortality After Percutaneous Coronary Intervention: Results From a Patient‐Level Pooled Analysis of the REPLACE‐2 (Randomized Evaluation of PCI Linking Angiomax to Reduced Clinical Events), ACUITY (Acute Catheterization and Urgent Intervention Triage Strategy), and HORIZONS‐AMI (Harmonizing Outcomes With Revascularization and Stents in Acute Myocardial Infarction) Trials,” JACC Cardiovascular Interventions 4 (2011): 654–664. 21700252 10.1016/j.jcin.2011.02.011

[3] D. N. Feldman , R. V. Swaminathan , L. A. Kaltenbach , et al., “Adoption of Radial Access and Comparison of Outcomes to Femoral Access in Percutaneous Coronary Intervention: An Updated Report From The National Cardiovascular Data Registry (2007‐2012),” Circulation 127 (2013): 2295–2306. 23753843 10.1161/CIRCULATIONAHA.112.000536

[4] G. Gargiulo , D. Giacoppo , S. S. Jolly , et al., “Effects on Mortality and Major Bleeding of Radial Versus Femoral Artery Access for Coronary Angiography or Percutaneous Coronary Intervention: Meta‐Analysis of Individual Patient Data From 7 Multicenter Randomized Clinical Trials,” Circulation 146 (2022): 1329–1343. 36036610 10.1161/CIRCULATIONAHA.122.061527

[5] H. Idris , J. K. French , I. M. Shugman , A. P. Hopkins , C. P. Juergens , and L. Thomas , “Influence of Age and Gender on Clinical Outcomes Following Percutaneous Coronary Intervention for Acute Coronary Syndromes,” Heart, Lung and Circulation 26 (2017): 554–565. 10.1016/j.hlc.2016.10.021 28034708

[6] J. Yu , R. Mehran , L. Grinfeld , et al., “Sex‐Based Differences in Bleeding and Long Term Adverse Events After Percutaneous Coronary Intervention for Acute Myocardial Infarction: Three Year Results From the HORIZONS‐AMI Trial,” Catheterization and Cardiovascular Interventions 85 (2015): 359–368. 25115966 10.1002/ccd.25630

[7] Y. Numasawa , S. Kohsaka , H. Miyata , et al., “Gender Differences in In‐Hospital Clinical Outcomes After Percutaneous Coronary Interventions: An Insight From a Japanese Multicenter Registry,” PLoS One 10 (2015): e0116496. 25635905 10.1371/journal.pone.0116496 PMC4312045

[8] R. C. Chester , S. A. Mina , B. Lewis , N. Zhang , R. Butterfield , and E. H. Yang , “Radial Artery Access Is Under‐Utilized in Women Undergoing PCI Despite Potential Benefits: Mayo Clinic PCI Registry,” Catheterization and Cardiovascular Interventions 95 (2020): 675–683. 31115141 10.1002/ccd.28341

[9] M. A. Essibayi , H. Cloft , L. E. Savastano , and W. Brinjikji , “Safety and Efficacy of Angio‐Seal Device for Transfemoral Neuroendovascular Procedures: A Systematic Review and Meta‐Analysis,” Interventional Neuroradiology 27 (2021): 703–711. 33601976 10.1177/1591019921996100 PMC8493339

[10] E. Nikolsky , R. Mehran , A. Halkin , et al., “Vascular Complications Associated With Arteriotomy Closure Devices in Patients Undergoing Percutaneous Coronary Procedures: A Meta‐Analysis,” Journal of the American College of Cardiology 44 (2004): 1200–1209. 15364320 10.1016/j.jacc.2004.06.048

[11] M. Iannaccone , G. Saint‐Hilary , D. Menardi , et al., “Network Meta‐Analysis of Studies Comparing Closure Devices for Femoral Access After Percutaneous Coronary Intervention,” Journal of Cardiovascular Medicine 19 (2018): 586–596. 30045086 10.2459/JCM.0000000000000697

[12] NCDR CathPCI registry v4.4 Coder's Data Dictionary . Accessed May 17, 2022, https://www.ncdr.com/WebNCDR/docs/default-source/public-data-collection-documents/cathpci_v4_codersdictionary_4-4.pdf?sfvrsn1/4b84d368e_2.

[13] R. P. Kreutz , S. Phookan , H. Bahrami , et al., “Femoral Artery Closure Devices vs Manual Compression During Cardiac Catheterization and Percutaneous Coronary Intervention,” Journal of the Society for Cardiovascular Angiography & Interventions 1 (2022): 100370. 39131476 10.1016/j.jscai.2022.100370 PMC11308787

[14] S. L. Daugherty , S. Kim , L. Thompson , et al., “Gender and Bleeding Risk Following Percutaneous Coronary Interventions: A Contemporary Report From the NCDR,” Journal of the American College of Cardiology 59 (2012): E1803‐E1803.

[15] B. Ahmed , W. D. Piper , D. Malenka , et al., “Significantly Improved Vascular Complications Among Women Undergoing Percutaneous Coronary Intervention: A Report From the Northern New England Percutaneous Coronary Intervention Registry,” Circulation: Cardiovascular Interventions 2 (2009): 423–429. 20031752 10.1161/CIRCINTERVENTIONS.109.860494

[16] V. Sharma , W. Wilson , W. Smith , et al., “Comparison of Characteristics and Complications in Men Versus Women Undergoing Chronic Total Occlusion Percutaneous Intervention,” American Journal of Cardiology 119 (2017): 535–541. 27923460 10.1016/j.amjcard.2016.11.004

[17] S. M. Gewalt , S. M. Helde , T. Ibrahim , et al., “Comparison of Vascular Closure Devices Versus Manual Compression After Femoral Artery Puncture in Women: Gender‐Based Analysis of a Large Scale, Randomized Clinical Trial,” Circulation Cardiovascular Interventions 11 (2018): e006074. 30354782 10.1161/CIRCINTERVENTIONS.117.006074

[18] V. Farooq , D. Goedhart , P. Ludman , et al., “Relationship Between Femoral Vascular Closure Devices and Short‐Term Mortality From 271 845 Percutaneous Coronary Intervention Procedures Performed in the United Kingdom Between 2006 and 2011: A Propensity Score–Corrected Analysis From the British Cardiovascular Intervention Society,” Circulation: Cardiovascular Interventions 9 (2016): e003560. 27225421 10.1161/CIRCINTERVENTIONS.116.003560

[19] G. Schnyder , N. Sawhney , B. Whisenant , S. Tsimikas , and Z. G. Turi , “Common Femoral Artery Anatomy Is Influenced by Demographics and Comorbidity: Implications for Cardiac and Peripheral Invasive Studies,” Catheterization and Cardiovascular Interventions 53 (2001): 289–295. 11458402 10.1002/ccd.1169

[20] Y. Lv , S. Zhang , X. Weng , et al., “Estrogen Deficiency Accelerates Postmenopausal Atherosclerosis by Inducing Endothelial Cell Ferroptosis Through Inhibiting NRF2/GPX4 Pathway,” FASEB Journal 37 (2023): e22992. 37219513 10.1096/fj.202300083R

[21] X. Li , W. Chen , P. Li , et al., “Follicular Stimulating Hormone Accelerates Atherogenesis by Increasing Endothelial VCAM‐1 Expression,” Theranostics 7 (2017): 4671–4688. 29187895 10.7150/thno.21216 PMC5706091

[22] Q. Meng , Y. Li , T. Ji , et al., “Estrogen Prevent Atherosclerosis by Attenuating Endothelial Cell Pyroptosis via Activation of Estrogen Receptor α‐Mediated Autophagy,” Journal of Advanced Research 28 (2021): 149–164. 33364052 10.1016/j.jare.2020.08.010 PMC7753237

[23] K. Mazurek , P. Zmijewski , A. Czajkowska , and G. Lutosławska , “Gender Differences in Carotid Artery Intima‐Media Thickness and Flow‐Mediated Dilatation in Young, Physically Active Adults,” Journal of Sports Medicine and Physical Fitness 54 (2014): 298–306. 24739292

[24] J. M. Aldrighi , R. L. S. Oliveira , É. D'Amico , et al., “Platelet Activation Status Decreases After Menopause,” Gynecological Endocrinology 20 (2005): 249–257. 16019369 10.1080/09513590500097549

[25] M. Notelovitz , C. S. Kitchens , V. Rappaport , L. Coone , and M. Dougherty , “Menopausal Status Associated With Increased Inhibition of Blood Coagulation,” American Journal of Obstetrics and Gynecology 141 (1981): 149–152. 6974498 10.1016/s0002-9378(16)32582-0

[26] H. Eggebrecht , C. von Birgelen , C. Naber , et al., “Impact of Gender on Femoral Access Complications Secondary to Application of a Collagen‐Based Vascular Closure Device,” Journal of Invasive Cardiology 16 (2004): 247–250. 15152129

[27] R. J. Applegate , M. Sacrinty , M. A. Kutcher , et al., “Vascular Complications With Newer Generations of Angioseal Vascular Closure Devices,” Journal of Interventional Cardiology 19 (2006): 67–74. 16483343 10.1111/j.1540-8183.2006.00107.x

[28] D. Scheinert , H. Sievert , M. A. Turco , et al., “The Safety and Efficacy of an Extravascular, Water‐Soluble Sealant for Vascular Closure: Initial Clinical Results for Mynx™,” Catheterization and Cardiovascular Interventions 70 (2007): 627–633. 17960627 10.1002/ccd.21353

[29] N. R. Holm , B. Sindberg , M. Schou , et al., “Randomised Comparison of Manual Compression and Femosealª Vascular Closure Device for Closure After Femoral Artery Access Coronary Angiography: The CLOsure dEvices Used in everyday Practice (CLOSE‐UP) Study,” EuroIntervention 10 (2014): 183–190. 24603054 10.4244/EIJV10I2A31

[30] E. Noory , T. Böhme , L. Krause , et al., “Evaluation of the MYNX CONTROL™ Arterial Closure System for Achieving Primary Hemostasis After Arterial Femoral Access Following Peripheral Arterial Interventions, Compared to the Femoseal Closure System,” Journal of Clinical Medicine 12 (2023): 5255. 37629297 10.3390/jcm12165255 PMC10455888

[31] J. H. Hwang , S. W. Park , W. Y. Yang , et al., “Safety and Efficacy of Mynx Vascular Closure Device for the Closure of Common Femoral Artery Access After Ipsilateral Stent Placement,” Journal of Vascular Access 23 (2022): 24–31. 33183180 10.1177/1129729820966946

[32] U. Gerckens , N. Cattelaens , E. G. Lampe , and E. Grube , “Management of Arterial Puncture Site After Catheterization Procedures: Evaluating a Suture‐Mediated Closure Device,” American Journal of Cardiology 83 (1999): 1658–1663. 10392872 10.1016/s0002-9149(99)00174-5

[33] R. G. Carere , J. G. Webb , T. Ahmed , and A. A. Dodek , “Initial Experience Using Prostar™: A New Device for Percutaneous Suture‐Mediated Closure of Arterial Puncture Sites,” Catheterization and Cardiovascular Diagnosis 37 (1996): 367–372. 8721692 10.1002/(SICI)1097-0304(199604)37:4<367::AID-CCD5>3.0.CO;2-9

[34] D. S. Baim , W. D. Knopf , T. Hinohara , et al., “Suture‐Mediated Closure of the Femoral Access Site After Cardiac Catheterization: Results of the Suture to Ambulate and Discharge (STAND I and STAND II) Trials,” American Journal of Cardiology 85 (2000): 864–869. 10758928 10.1016/s0002-9149(99)00882-6

[35] L. Zornitzki , D. Zahler , S. Frydman , et al., “Vascular Complications in Transcatheter Aortic Valve Replacement With Plug‐Based vs Suture‐Based Closure Devices,” Canadian Journal of Cardiology 39 (2023): 1528–1534. 37419247 10.1016/j.cjca.2023.06.425

